# Hepatitis B virus X protein inhibits extracellular IFN-α-mediated signal transduction by downregulation of type I IFN receptor

**DOI:** 10.3892/ijmm.2012.879

**Published:** 2012-01-03

**Authors:** IL-RAE CHO, MYUNGJU OH, SANG SEOK KOH, WARAPORN MALILAS, RATAKORN SRISUTTEE, BYUNG HAK JHUN, SANDRA PELLEGRINI, SERGE Y. FUCHS, YOUNG-HWA CHUNG

**Affiliations:** 1 WCU Department of Cogno-Mechatronics Engineering, BK21 Nanofusion Technology Team, Pusan National University, Busan 609-735; 2 WCU Department of Nanomedical Engineering, BK21 Nanofusion Technology Team, Pusan National University, Busan 609-735; 3 Therapeutic Antibody Research Center, Korea Research Institute of Bioscience and Biotechnology, Daejeon 305-333, Republic of Korea; 4 Cytokine Signalling Unit, CNRS URA1961 Pasteur Institute, Paris 75724, France; 5 Department of Animal Biology and Mari Lowe Center for Comparative Oncology Research, University of Pennsylvania, Philadelphia, PA 19104, USA

**Keywords:** hepatitis B virus X, IFN-α receptor, Tyk2, IFN-α signaling

## Abstract

We have previously shown that hepatitis B virus (HBV) protein X (HBX), a regulatory protein of HBV, activates Stat1, leading to type I interferon (IFN) production. Type I IFN secreted from HBX-expressing hepatic cells enforces antiviral signals through its binding to the cognate type I IFN receptor. We therefore investigated how cells handle this detrimental situation. Interestingly, compared to Chang cells stably expressing an empty vector (Chang-Vec), Chang cells stably expressing HBX (Chang-HBX) showed lower levels of IFN-α receptor 1 (IFNAR1) protein, a subunit of type I IFN receptor. The levels of IFNAR1 transcripts detected in Chang-HBX cells were lower than the levels in Chang-Vec cells, indicating that HBX regulates IFNAR1 at the transcriptional level. Moreover, we observed that HBX induced the translocation of IFNAR1 to the cytoplasm. Consistent with these observations, HBX also downregulated Tyk2, which is required for the stable expression of IFNAR1 on the cell surface. Eventually, Chang-HBX cells consistently maintained a lower level of IFNAR1 expression and displayed no proper response to IFN-α, while Chang-Vec cells exhibited a proper response to IFN-α treatment. Taken together, we propose that HBX downregulates IFNAR1, leading to the avoidance of extracellular IFN-α signal transduction.

## Introduction

The type I interferon (IFN) receptor consists of 2 subunits, IFN-α receptor 1 (IFNAR1) and IFNAR2, which belong to the type II cytokine receptor superfamily (1). This heterodimeric complex is able to interact with IFN-α and IFN-β, resulting in the phosphorylation of Tyk2 and Jak1 that are bound to IFNAR1 and IFNAR2, respectively (2). Subsequently, the Stat proteins, Stat1 and Stat2 are phosphorylated at specific tyrosine residues, which allows the 2 proteins to form a Stat1/2 heterodimer based on SH2/phosphotyrosine interactions. The formation of this heterodimer facilitates its association with IFN regulatory factor (IRF)9 to form an active heterotrimeric transcription factor called IFN-stimulated gene factor (ISGF)3. ISGF3 targets specific sequences such as IFN-stimulated response element and IFN-γ-activated sequence in the promoters of IFN-stimulated genes, leading to the establishment of antiviral status (3).

After type I IFN binds to its cognate type I IFN receptor, the receptor is downregulated by endocytosis mediated by cargo-specific clathrin machinery, and degraded via the lysosomal and proteosomal pathways in order to limit the magnitude and duration of IFN signaling (4,5). The adaptin protein 2 complex, a component of the cargo-specific clathrin machinery recognizes the Tyr-based endocytic motif of IFNAR1, leading to the efficient endocytosis of IFNAR1 (5). The Skip, Cullin, F-box containing complex β-TrCP E3 ubiquitin ligase mediates the ubiquitination of IFNAR1 in a phosphorylation-dependent manner, eventually designating IFNAR1 for lysosomal degradation (5). Catalytic activation of Tyk2 is required for these events but is not essential for IFNAR1 internalization (6). Conversely, it has been also reported that Tyk2 is essential for the stable cell surface expression of IFNAR1 and stabilizes IFNAR1 by its interaction in the basal condition (in the absence of ligand) (7). Further studies have revealed that binding of Tyk2 in the proximity of the Tyr-based linear motif of IFNAR1 is required to prevent IFNAR1 internalization and to maintain its cell surface expression by physically shielding the Tyr-based motif from recognition by AP2, a component of the endocytic cargo machinery (8).

The human hepatitis B virus (HBV) induces acute and chronic hepatitis and is closely associated with the incidence of human liver cancer (9). Among the 4 proteins that are derived from the HBV genome, the hepatitis B virus X (HBX) protein is involved in multiple signaling pathways associated with cell survival and proliferation. Cell signal transduction pathways that are activated by HBX include the Jak1/Stat3, PI-3 kinase pathways (10–13), and the Ras/Raf/MAPK signaling cascade which leads to NF-κB activation (14,15). HBX expression also increases reactive oxygen species via calcium signaling and cellular kinases, resulting in the activation of transcription factors NF-κB and Stat3 (10). Studies have revealed that HBV-induced oxidative stress also stimulates the translocation of Raf-1. Src inhibitors or a dominant negative PAK mutant abolishes HBX-mediated Raf-1 mitochondrial translocation (16). Recently, we have shown that HBX-mediated up-regulation of Foxo4 plays a critical role in the prevention of oxidative stress-induced apoptosis in a liver cell line (17).

Based on our observation that HBX induces the production of type I IFN by the activation of Stat1 (18), we believed it is likely that secreted type I IFN from HBX-expressing hepatic cells enforces antiviral signals through its binding to the cognate type I IFN receptor. We initiated this study to investigate how HBX-expressing hepatic cells overcome this unfavorable situation. Here, we reported that HBX expression downregulates type I IFN receptor, leading to disturbance of extracellular type I IFN signaling.

## Materials and methods

### Cell cultures, reagent and antibodies

Chang cells, Chang cells stably expressing vector (Chang-Vec), Chang cells stably expressing HBX (Chang-HBX), and HEK 293 T cells were cultured in DMEM supplemented with 10% FBS and 1% penicillin and streptomycin. IFN-α was purchased from R&D Systems (Minneapolis, MN). Anti-IFNAR1 and anti-IFNγR and rabbit polyclonal anti-c-Myc antibodies were purchased from Abcam (Cambridge, MA) and β-tubulin antibodies were obtained from Santa Cruz Biotechnology, Inc. (Santa Cruz, CA). Antibodies against Tyk2 and Jak1 were acquired from Cell Signaling (Danvers, MA) and anti-Flag antibody was obtained from Sigma-Aldrich (St. Louis, MO).

### siRNA transfection

Cells were trypsinized and incubated overnight to achieve 60–70% confluence before siRNA transfection. Tyk2 siRNA (60 nM, sense 5′-UCUCACCUCUUCC CAUUCC(dTdT)-3′ and antisense 5′-GGAAUGGGAAGAGGU GAGA(dTdT)-3′) purchased from Bioneer (Daejeon, Korea) or control siRNA (19) were mixed with Lipofectamine 2000 (Invitrogen, Carlsbad, CA). The cells were incubated with the transfection mixture for 6 h and then rinsed with DMEM containing 10% serum. The cells were incubated for 48 h before harvest.

### Western blotting

Cells were harvested and treated with lysis buffer (150 mM NaCl, 1% NP-40, 50 mM Tris-HCl pH 7.5) containing 0.1 mM Na_2_VO_3_, 1 mM NaF and protease inhibitors (Sigma-Aldrich). For immunoblotting, proteins from whole cell lysates were resolved by 10 or 12% SDS-PAGE and then transferred to nitrocellulose membranes. Primary antibodies were used at 1:1,000 or 1:2,000 dilutions, and secondary antibodies conjugated with horseradish peroxidase were used at 1:2,000 dilutions in 5% nonfat dry milk. After a final wash, nitrocellulose membranes were exposed for an enhanced chemiluminescence assay using LAS 3000 (Fuji, Tokyo, Japan).

### Reverse transcription-polymerase chain reaction (RT-PCR) analysis

Total-RNA was extracted from the cells using the RNeasy micro kit (Qiagen, Valencia, CA) in accordance with the manufacturer’s instructions. Three micrograms of total RNA were converted to cDNA using Superscript II reverse transcriptase (Invitrogen), and PCR was performed using specific primers described elsewhere (20). The cDNAs of each sample were diluted, and PCR was run at the optimized cycle number. β-actin mRNA was measured as an internal standard. After amplification, the products were subjected to electrophoresis on 1.5% agarose and detected by ethidium bromide staining.

### Immunofluorescence

Cells were fixed with 4% paraformaldehyde for 15 min, permeabilized with cold acetone for 15 min, blocked with 10% goat serum for 30 min, and reacted with a 1:100-diluted primary antibody for 30 min at room temperature. After incubation, the cells were washed extensively with PBS, incubated with a 1:500-diluted Alexa Fluor 680-conjugated goat anti-rabbit IgG antibody (Molecular Probes, Eugene, OR), or with a 1:500-diluted Alexa Fluor 514-conjugated goat anti-mouse IgG antibody (Molecular Probes) in PBS for 30 min at room temperature, and then washed 3 times with PBS. The stained cells were mounted with PBS containing 10% glycerol and photographed using a LSM510 confocal microscope (Zeiss, Oberkochen, Germany).

## Results

### HBX expression induces downregulation of type I IFN-α receptor 1

**Figure 1:**
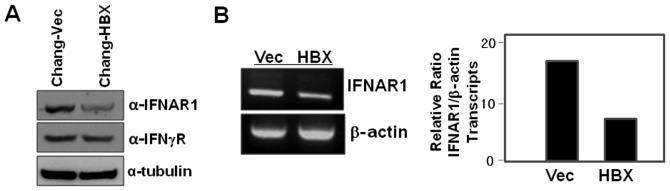
HBX downregulates IFNAR1 at the transcriptional level. (A) Cell lysates of Chang-Vec and Chang-HBX cells were electrophoretically separated on 10% SDS-PAGE. IFNAR1 and IFNγR were detected with the corresponding antibodies. (B) Total-RNA (3 μg) isolated from Chang-Vec and Chang-HBX cells was subjected to RT-PCR. The IFNAR1 sequence was amplified using its specific primers and visualized on a 1.5% agarose gel by ethidium bromide staining. β-actin was used as an internal control.

We have previously shown that HBX expression mimics intracellular type I IFN signaling through Stat1 activation in Chang cells, leading to the production and secretion of type I IFN into the surrounding microenvironment (18). Although we previously proposed that HBX may protect HBV-infected hepatic cells from lytic infection of viruses, we now face a contradiction; the delivery of an enforced antiviral signal to the cells by the released type I IFN would exert a disadvantageous effect on HBX-expressing hepatic cells. How do the HBX-expressing hepatic cells resolve this detrimental situation? Downregulation of the type I IFN receptor may well solve this contradiction. To test our hypothesis, we examined whether the type I IFN receptor consisting of 2 subunits; IFNAR1 and IFNAR2 is downregulated in the presence of HBX. Interestingly, we found that IFNAR1 is less abundant in the cell lysates of Chang-HBX cells than in those of Chang-Vec cells (Fig. 1A). We next examined IFN-γ receptor levels in both Chang-HBX and Chang-Vec cells, and found that the levels of IFN-γ receptor in Chang-HBX cells are similar to those in Chang-Vec cells (Fig. 1A).

To explore how IFNAR1 is downregulated in the presence of HBX (Fig. 1A), we examined IFNAR1 transcript levels in Chang-Vec and Chang-HBX cells to assess HBX-mediated transcriptional regulation. The abundance of IFNAR1 transcripts in Chang-HBX cells was lesser than that in Chang-Vec cells (Fig. 1B). This indicates that HBX might play an important role in the transcriptional regulation of IFNAR1.

### HBX induces translocation of IFNAR1 into the cytoplasm

**Figure 2:**
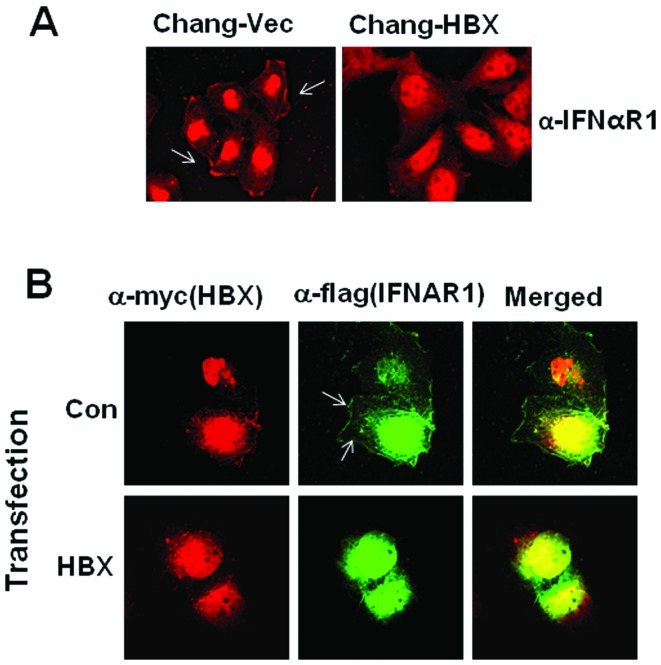
HBX induces translocation of IFNAR1 into the cytoplasm. (A) Chang-Vec and Chang-HBX cells were fixed with 4% paraformaldehyde and permeabilized with cold acetone. The cells were incubated with anti-IFNAR1 and visualized by confocal microscopy after staining with Alexa Fluor 680-conjugated goat anti-rabbit IgG antibody. (B) Chang cells were transfected with a Flag-tagged IFNAR1 vector with or without a Myc-tagged HBX vector and fixed 2 days after transfection. The cells were incubated with rabbit polyclonal c-Myc antibodies (HBX) and mouse monoclonal anti-Flag antibody and visualized by confocal microscopy after staining with Alexa Fluor 680-conjugated goat anti-rabbit IgG antibody together with Alexa Fluor 514-conjugated goat anti-mouse IgG antibody.

In addition to the HBX-mediated decrease in IFNAR1 expression, another possible mechanism for the efficient blockage of type I IFN receptor-mediated antiviral signaling is translocation of the IFN receptor into the cytoplasm. To test this theory, we attempted to examine IFNAR1 localization after staining using confocal microscopy. We found that IFNAR1 is localized in the cytoplasm of Chang-HBX cells, but preferentially localized in the plasma membrane of Chang-Vec cells (Fig. 2A). To confirm the cytosolic localization of IFNAR1 in the presence of HBX, exogenous IFNAR1 tagged with a Flag epitope (IFNAR1-Flag) was employed with a Myc-tagged HBX expression vector. IFNAR1-Flag was detected in the cytosol rather than in the membrane in the presence of HBX, similarly to endogenous IFNAR1 in Chang-HBX cells (Fig. 2B). On the other hand, in the absence of HBX, exogenous IFNAR1 in Chang cells was found in the plasma membrane similar to endogenous IFNAR1 in Chang-Vec cells (Fig. 2B).

### Decrease of Tyk2 mediated by HBX diminishes IFNAR1 levels

**Figure 3:**
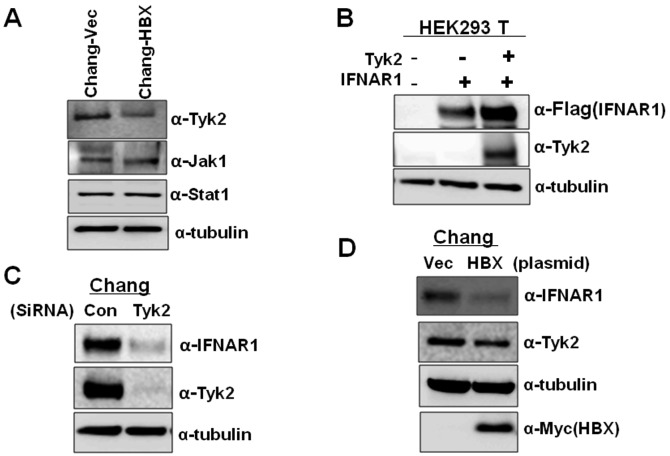
HBX-mediated decrease of Tyk2 causes a reduction in IFNAR1 levels. (A) Cell lysates from Chang-Vec and Chang-HBX cells were electrophoretically separated on 10% SDS-PAGE. Tyk2, Jak1 and Stat1 were detected with the corresponding antibodies. (B) HEK293 T cells were transfected with a Flag-tagged IFNAR1 vector (1 μg) with or without a Tyk2 expression vector (1 μg) using the calcium phosphate precipitation method. Two days post-transfection, cells were harvested and the cell lysates separated on 10% SDS-PAGE followed by immunoblotting with anti-Flag and anti-Tyk2 antibodies. (C) Chang cells were transfected with Tyk2 siRNA (60 nM) and control siRNA (60 nM). Two days post-transfection, cells were harvested and the cell lysates were separated on 10% SDS-PAGE followed by immunoblotting with anti-IFNAR1 and anti-Tyk2 antibodies. (D) Chang cells were transfected with a Myc-tagged HBX vector (2 μg) and endogenous IFNAR1 and Tyk2 were detected by immunoblotting 2 days post-transfection.

Since previous studies have shown that Tyk2 is essential for the stable cell surface expression of IFNAR1 and stabilizes IFNAR1 by its interaction in the basal condition (in the absence of ligand) (7), we examined the expression levels of Tyk2 in Chang-Vec and Chang-HBX cells. The Chang-HBX cells exhibited a significantly lower abundance of Tyk2 than Chang-Vec cells (Fig. 3A). However, the levels of Jak1 were similar in both cells. Similar protein quantities of Stat1, which is associated with the IFN signaling pathway, were also found in both cells although highly activated Stat1 was found in Chang-HBX cells (18). To confirm that the level of Tyk2 determines the IFNAR1 protein level, we transiently expressed IFNAR1 alone or together with Tyk2 in HEK 293T cells. As seen in Fig. 3B, compared to the expression of IFNAR1 only, co-expression of IFNAR1 with Tyk2 enhanced the level of IFNAR1 expression. We also examined the effect of reduced expression of Tyk2 on IFNAR1 protein levels in Chang cells. When siRNA against Tyk2 was introduced into Chang cells expressing endogenous IFNAR1, expression of IFNAR1 was significantly reduced than that in Chang cells treated with control siRNA (Fig. 3C). To confirm that the presence of HBX downregulates the level of Tyk2 protein, which results in lower IFNAR1 expression, we transiently introduced HBX into Chang cells. We found that HBX downregulates the expression of Tyk2, leading to a decrease in IFNAR1 expression while control vector fails to decrease the Tyk2 level (Fig. 3D). These results indicate that HBX also modulates IFNAR1 expression via Tyk2.

### IFNAR1 does not function normally in Chang-HBX cells during IFN-α signaling

**Figure 4:**
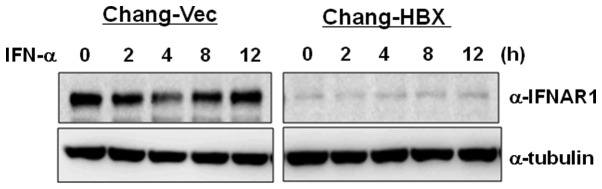
Chang-HBX cells display no alterations in IFNAR1 expression during exogenous IFN-α signaling. Chang-Vec and Chang-HBX cells were treated with IFN-α (1,000 U/ml) for 12 h. Cells were harvested and the cell lysates separated on 10% SDS-PAGE, followed by immunoblotting with anti-IFNAR1 antibody.

We have shown that the presence of HBX suppresses IFNAR1 transcription directly (Fig. 1B), and downregulates IFNAR1 expression via Tyk2 in the absence of its ligand; IFN-α (Fig. 3). To examine how IFNAR1 responds to type I IFN in Chang-Vec and Chang-HBX cells, both cell lines were treated with IFN-α for 12 h. IFNAR1 levels were reduced at 4 h post-treatment and then returned to original levels at 8 h post-treatment in Chang-Vec cells (Fig. 4). However, the reduced IFNAR1 level was not altered during IFN-α signaling for 12 h in Chang-HBX cells. This result indicates that IFNAR1 may not function normally in Chang-HBX cells during IFN-α signaling.

## Discussion

HBV infection afflicts more than 400 million people worldwide and accelerates the development of hepatocellular carcinoma (21). Regarding the role of HBX-mediated type I IFN production, we initially proposed that type I IFN inhibits super-infection by the virus, protecting the host and eventually maintaining chronic infection of HBV (18). We additionally propose that type I IFN mediated by HBX may play a role in inflammation which is believed to be closely related to liver carcinogenesis on the basis of mounting evidence from preclinical and clinical studies that persistent inflammation functions as a driving force in the development of cancer (22,23). However, the autocrine or paracrine effects of the released type I IFN from HBV-infected hepatic cells are yet unknown. Type I IFN enforces antiviral status through its binding to the cognate receptor; how do the HBV-infected hepatic cells respond to this unfavorable condition? In this study, we report the answer to this question; HBX downregulates IFNAR1, leading to avoidance of extracellular type I IFN-mediated signaling.

Several viruses have evolved strategies of immune evasion to impair type I IFN signaling pathways. The E6 protein of human papillomavirus (HPV) 18 has been shown to selectively interact with Tyk2 and block its activation (24). Japanese encephalitis virus infection also selectively impairs Tyk2 phosphorylation and West Nile virus infection hinders the phosphorylation of both Tyk2 and Jak1 (25,26); however, the mediators of this blockade are unknown in both cases. Other viruses affect immune escape by causing a blockage of IFN signaling at the level of Stat activation. The V protein of Sendai virus 5 and other paramyxoviruses target the Stat protein for degradation and the Sendai virus C protein interferes with Stat phosphorylation (27,28). In addition, the E7 protein of HPV impairs the assembly of IRF9 and the E1A protein of adenovirus impedes the interaction of Stat1 with transcriptional machinery (29,30). Recently, the hepatitis C virus has been shown to interfere with Stat1 activation by up-regulation of protein phosphatase 2A and the RIF protein of Kaposi’s sarcoma associated herpesvirus forms inhibitory complexes with several proteins including Tyk2, Jak1, Stat1 and Stat2, leading to the inhibition of Stat1 and Stat2 (31,32). Reviewing these lines of evidence, downregulation of Tyk2 mediated by HBX is a unique mechanism different from dephosphorylation of Tyk2 in Chang cells and, eventually leads to cytosolic localization of IFNAR1. In this study, we provide evidence of a novel mechanism for the modulation of type I IFN signaling by the downregulation of Tyk2. Related to regulation via Tyk2, another study has reported that expression of SHP-1 is diminished or abolished in most lymphoma cell lines and in some colorectal cancer (33–35). Conversely, transient expression of SHP-1 inhibits tumor cell growth via downregulation of Jak1 and Tyk2 (33). Our future study will be directed towards exploring whether protein phosphatases including SHP-1 are associated with the specific degradation of Tyk2. Type I IFN signaling pathway involves the binding of type I IFN to its receptor, and then phosphorylation of Jak1 and Tyk2 takes place followed by the activation of Stat1. We herein suggest that the phosphorylation of Stat1 is independent of Tyk2 at least in the Chang-HBX cells.

In addition, we also observed that IFNAR1 is regulated at the transcriptional level. It is possible that HBX induces a transcription factor with suppressor activity to bind to the promoter of IFNAR1 as seen in the case of PTEN promoter, which is occupied by the p53 tumor suppressor in the presence of HBX (36). HBX might cause an instability in IFNAR1 mRNA by inducing the release of a RNA-binding protein such as HuR from the 3′ untranslated region (37). Moreover, HBX may induce methylation at a G/C region in the IFNAR1 promoter via the upregulation of DNA methyltransferase (DNMT) 1 activity; HBX has similarly been reported to activate DNMT1, resulting in suppression of p16(INK1a), a cyclin-dependent kinase inhibitor through hypermethylation of the p16(INK1a) promoter (38). The detailed mechanism of HBX-mediated suppression of IFNAR1 at the transcriptional level remains under investigation.
